# Supplementary material for: Mucosal Plasma Cell Activation and Proximity to Nerve Fibres Are Associated with Glycocalyx Reduction in Diarrhoea-Predominant Irritable Bowel Syndrome: Jejunal Barrier Alterations Underlying Clinical Manifestations
Source: Cells. 2022 Jun 28;11(13):2046. doi: 10.3390/cells11132046 (PMC9265332; doi:10.3390/cells11132046)
Supplement: Supplementary file 1 [file cells-11-02046-s001.zip › cells-1772446-supplementary.pdf]

Supplementary material

Mucosal plasma cell activation and proximity to nerve fibres are associated with glycocalyx reduction in diarrhoea-predominant irritable bowel syndrome: jejunal barrier alterations underlying clinical manifestations.

Authors

Cristina Pardo-Camacho[1,2,3†] John-Peter Ganda Mall[1,4†], Cristina Martínez[5], Marc Pigrau[2,3,], Elba Expósito[1,2], Mercé Albert-Bayo[1], Elisa Melón-Ardanaz[1], Adoración Nieto[2,6], Bruno Rodiño-Janeiro[2], Marina Fortea[1], Danila Guagnozzi[1,2,6], Amanda Rodriguez-Urrutia[3,7,8], Inés de Torres[3,9], Ignacio Santos-Briones[10], Fernando Azpiroz[6,11], Beatriz Lobo[2,3,6], Carmen Alonso-Cotoner[2,3,6,11], Javier Santos[2,3,6,11], Ana M González-Castro[1,2†*], Maria Vicario[1,12†*]

*Corresponding authors

[†]Authors contributed equally.

[1]Translational Mucosal Immunology Group, Digestive System Research Unit, Vall d'Hebron Institut de Recerca (VHIR), Passeig Vall d'Hebron 119-129, 08035 Barcelona, Spain. [2]Neuro-Immuno-Gastroenterology Group, Digestive System Research Unit, Vall d'Hebron Institut de Recerca (VHIR), Passeig Vall d'Hebron 119-129, 08035 Barcelona, Spain. [3]Facultat de Medicina, Universitat Autònoma de Barcelona, 08193 Bellaterra, Spain. [4]Department of Biomedical and Clinical Sciences, Linköping University, Linköping, Sweden. [5]Vascular and Renal Translational Research Group, Lleida Institute for Biomedical Research Dr. Pifarré. Foundation (IRBLleida), Av. Alcalde Rovira Roure 80, 25198 Lleida, Spain. [6]Department of Gastroenterology, Vall d'Hebron Hospital Universitari, Passeig Vall d'Hebron 119-129, 08035 Barcelona, Spain. [7]Department of Mental Health, Vall d'Hebron Hospital Universitari, Passeig Vall

d'Hebron 119-129, 08035 Barcelona, Spain. [8]Centro de Investigación Biomédica en Red de Salud Mental (CIBERSAM), Instituto de Salud Carlos III, 28029 Madrid, Spain. [9]Department of Pathology, Vall d'Hebron Hospital Universitari, Passeig Vall d'Hebron 119-129, 08035 Barcelona, Spain. [10]Universitat Ramon LLull-Blanquerna, Facultat Ciències de la Salut, C/Padilla 326, 08025, Barcelona. [11]Centro de Investigación Biomédica en Red de Enfermedades Hepáticas y Digestivas (CIBEREHD), Instituto de Salud Carlos III, 28029 Madrid, Spain. [12]Department of Gastrointestinal Health, Nestlé Institute of Health Sciences, Société des Produits Nestlé S.A. Nestlé Research, Vers-chez-les-Blanc, 1000 Lausanne, Switzerland.

Corresponding authors:

Ana M González-Castro PhD, Neuro-Immuno-Gastroenterology Group, Digestive System Research Unit, Vall d'Hebron Institut de Recerca (VHIR), Vall d'Hebron Hospital Universitari, Passeig Vall d'Hebron 119-129, 08035 Barcelona, Spain; e-mail: ana.maria.gonzalez@vhir.org

María Vicario PhD, Translational Mucosal Immunology Group, Digestive System Research Unit, Vall d'Hebron Institut de Recerca (VHIR), Vall d'Hebron Hospital Universitari, Passeig Vall d'Hebron 119-129, 08035 Barcelona, Spain; e-mail: maria.vicario@vhir.org
Department of Gastrointestinal Health, Nestlé Institute of Health Sciences, Société des Produits Nestlé S.A. Nestlé Research, Vers-chez-les-Blanc, 1000 Lausanne, Switzerland. email: maria.vicarioperez@rd.nestle.com

A. Supplementary methods

Table S1. Inclusion and exclusion criteria of the study

	HC	IBS-D
**Inclusion criteria**		
Age: 18-60 years	yes	yes
Rome III criteria for IBS-D	no	yes
Naïve (newly-diagnosed)	no	yes
Acceptance of the study protocol	yes	yes
**Exclusion criteria**		
Clinical history of food allergy	yes	yes
Positivity to SPT to foodstuff	yes	yes
Infectious gastroenteritis	yes	yes
Gastrointestinal comorbidities	yes	yes
Pregnancy	yes	yes
Major psychiatric disorders	yes	yes
Abnormal mucosal histology	yes	yes
Use of medication (steroids, immunosuppressive drugs, anti-histaminic and mast cell stabilizers)	yes	yes

HC: Healthy control**;** IBS-D: Diarrhoea-predominant Irritable Bowel Syndrome; SPT: skin prick test.

Quantification of plasma cell activation

The quantitative assessment of plasma cell activation was performed in several steps: The captured TEM images of whole plasma cells were first imported into the ImageJ software application (v1.52, NIH, USA) to perform standardized measurements according to the scale bars of each image. This was followed by manually drawing the full cell outline and nuclei and thereby using the built-in tools of the software to calculate the area. The number and area of mitochondria were manually counted and measured, respectively, and both results are expressed as the average number per plasma cell. For quantifying the membrane area of the rough endoplasmic reticulum (RER), the images were first loaded into Adobe Photoshop CS2 (CA, USA) and then had both the surrounding environment outside the plasma cells and pixel-dense objects within, such as mitochondria and nuclei, manually removed to not interfere with the RER measurement. The edited images were further imported into ImageJ, scaled and then had the RER masked using the threshold tool of the ImageJ software. The masked area was finally used for calculating the membrane area of the RER. To calculate the %RER in the cytoplasm, as a surrogate marker of plasma cell activation, the nuclei area was first subtracted from the total cell area to obtain the area of the cytoplasm. The RER area was then divided by the cytoplasm area and multiplied by 100 to show the %RER in cytoplasm. This method was validated by comparing the results obtained in plasma cells from healthy subjects from Kirk *et al.*[1], with comparable findings being obtained (Table S3).

Table S2: Antibodies and conditions used for identification of protein expression by immunofluorescence in jejunal biopsies from HC and IBS-D patients.

**Primary antibody**				
**Antibody**	**Target**	**Supplier**	**Permeabilization**	**Incubation & Dilution**
**Rabbit anti-CD138**	Plasma cell	Sigma-Aldrich	0.1% Triton X-100	1:100 1h
**Mouse anti-PGP9.5**	Neuron	Abcam		1:50 O/N
**Secondary antibody**				
**Antibody**	**Marker**	**Supplier**		**Incubation & Dilution**
**Goat Alexa Fluor 594**	Anti-rabbit	ThermoFisher		1:500 30 min
**Goat Alexa Fluor 488**	Anti-mouse	ThermoFisher		

O/N: over-night incubation.

Table S3. Comparison of validated plasma cell activation markers by Kirk *et al*.[1], with the results obtained in our HC cohort.

	**RER/µm²**	**Nucleus area/µm²**	**Total cell area/µm²**	**Cytoplasm area/µm²**	**% RER in PC**
Kirk *et al*	3.36 ± 0.66	10.18 ± 1.1	28.23 ± 3.36	18.05 ± 2.62	18.21 ± 4.85
HC (n=11)	4.32 ± 1.07	11.6 ± 3.78	33.8 ± 8.28	22.3 ± 5.04	19.6 ± 3.26

The first line of data displays the reference results from a 7-day differentiated B cells (isolated from blood) into plasma cells. The 2nd line displays our results based on quantification of jejunal mucosal plasma cells from a total of 11 HCs. Results are expressed as mean±SD. [1]Kirk, S.J.; Cliff, J.M.; Thomas, J.A.; Ward, T.H. Biogenesis of secretory organelles during B cell differentiation. J Leukoc Biol 2010, 87, 245-255, doi:10.1189/jlb.1208774.

Table S4. Ig assays used for the Ig quantification in stool samples.

**Assay**	**Manufacturer**
Human IgAs ELISA kit	Immunodiagnostik
Human IgM ELISA kit	Bethyl Laboratories
Human IgG ELISA kit	Bethyl Laboratories
Human IgG1 ELISA kit	Ray Biotech
Human IgG2 ELISA kit	Abcam
Human IgG3 ELISA kit	Abnova
Human IgG4 ELISA kit	Cloud-Immunoassay
Human IgE ELISA kit	Bethyl Laboratories

B. Supplementary results

Quality control of the samples for RNA seq analysis

Three out of the 40 samples were discarded for DGE analysis based on the exploratory analysis result. The final dataset included 18 samples from IBS-D and 19 samples from HC. All samples were successfully aligned to the human reference genome (hg38) showing between 84.3% and 94.1% uniquely mapped reads. A complete gene annotation matrix was obtained from the .gtf annotation file which referred to 60,721 different genes by an Ensembl ID. Reads were mainly distributed in exons (coding sequence exons 61.1%-75.7%). Therefore, the percentage of assigned reads for gene quantification (counts matrix) was high (87.2% - 94%). Those genes with less than 10 reads among all samples were removed from the analysis. This led to a final raw counts matrix referring to 27,833 genes.

Table S5. Analysis of age, and clinical and histological variables stratified by sex in the IBS-D group.

Variables	**Women** (n=49)	**Men** (n=21)	***p***
**Age**	35.5 (18.0-65.0)	37.0 (20.0-48.0)	0.778
**Intensity of abdominal pain, score**	54.0 (10.0-100.0)	31.0 (2.0-80.0)	**0.050**
**Frequency of abdominal pain**	5.0 (1.0-10.0)	4.0 (0.0-10.0)	0.530
**Bowel movements**, number/day	3.2 (0.5-10.0)	3.0 (1.0-12.0)	0.430
**Stool form**, Bristol score	6.0 (3.0-7.0)	5.5 (3.0-7.0)	0.690
**Dyspepia** (%) *	71.4%	26.4%	**0.001**
**IBS-SSS**	288.0 (80.0-459.0)	221.0 (110.0-384.0)	**0.008**
**Holmes-Rage scale**, score	119.0 (0.0-889.0)	109.0 (15.0-318.0)	0.730
**Cohen scale**, score	25.0 (11.0-41.0)	18.0 (1.0-36.0)	**0.008**
**Beck's Depression Inventory**, score	9.5 (1.0-31.0)	5.0 (1.0-17.0)	**0.038**
**$CD117^+$** (cells/hpf)	22.2 (5.6-49.3)	30.6 (7.6-58.7)	**0.042**
**$CD3^+$** (cells/100 enterocytes)	17.0 (1.6-64.0)	16.0 (6.0-33.7)	0.826
**$Eosin^+$** (cells/hpf)	1.5 (0.0-15.20)	2.6 (0.0-17.7)	**0.018**

*Fischer's exact test used for assessing statistical difference between proportions.

Table S6. Description and enrichment score (ES) of the top 50 positive and top 50 negative enriched genes correlated to the IBS-D phenotype identified by GSEA.

Gene Name	Description	ES
GREM1	gremlin 1, DAN family BMP antagonist	0.35764793
IGKV1D-16	immunoglobulin kappa variable 1D-16	0.28704396
GREM2	gremlin 2, DAN family BMP antagonist	0.28564823
GGT4P	gamma-glutamyltransferase 4 pseudogene	0.26110834
IGHV3-73	immunoglobulin heavy variable 3-73	0.22686873
IGLV8-61	immunoglobulin lambda variable 8-61	0.2262599
IGLV3-9	immunoglobulin lambda variable 3-9	0.22415051
GPR15	G protein-coupled receptor 15	0.22363463
ERAP2	endoplasmic reticulum aminopeptidase 2	0.22044125
IGLV7-46	immunoglobulin lambda variable 7-46	0.21653804
TYRP1	tyrosinase related protein 1	0.2101466
MT-TT	mitochondrially encoded tRNA-Thr (ACN)	0.20960961
IGKV1D-13	immunoglobulin kappa variable 1D-13	0.20842642
IGLV2-18	immunoglobulin lambda variable 2-18	0.2061328
IGLV3-21	immunoglobulin lambda variable 3-21	0.20523998

IGLV5-45	immunoglobulin lambda variable 5-45	0.20251097
RPL13P12	ribosomal protein L13 pseudogene 12	0.19838658
IGKV6D-21	immunoglobulin kappa variable 6D-21	0.19504908
IGHG3	immunoglobulin heavy constant gamma 3 (G3m marker)	0.19385576
PSPHP1	phosphoserine phosphatase pseudogene 1	0.19355305
HPCAL4	hippocalcin like 4	0.18744418
IGHV3-20	immunoglobulin heavy variable 3-20	0.18649891
CCL13	C-C motif chemokine ligand 13	0.18555976
COL14A1	collagen type XIV alpha 1 chain	0.18538634
IGLV2-11	immunoglobulin lambda variable 2-11	0.18495513
IGKV1-16	immunoglobulin kappa variable 1-16	0.18327112
IGKV1-9	immunoglobulin kappa variable 1-9	0.18130335
IGHV3-49	immunoglobulin heavy variable 3-49	0.18070081
IGHV4-61	immunoglobulin heavy variable 4-61	0.18058859
IGHV1-58	immunoglobulin heavy variable 1-58	0.18039118
SFRP2	secreted frizzled related protein 2	0.17968023
IGLV1-51	immunoglobulin lambda variable 1-51	0.17628723
IGHV3-74	immunoglobulin heavy variable 3-74	0.17606895
CDA	cytidine deaminase	0.1748696

UGT1A4	UDP glucuronosyltransferase family 1 member A4	0.1735264
IGLV3-27	immunoglobulin lambda variable 3-27	0.17339191
IGLV2-8	immunoglobulin lambda variable 2-8	0.17269543
LCN2	lipocalin 2	0.17171861
IGLV1-41	immunoglobulin lambda variable 1-41	0.17142034
IGHV3-30	immunoglobulin heavy variable 3-30	0.16960447
ACOT12	acyl-CoA thioesterase 12	0.16855735
IGHG2	immunoglobulin heavy constant gamma 2 (G2m marker)	0.16839889
IGKV1-6	immunoglobulin kappa variable 1-6	0.16681471
IGHV7-4-1	immunoglobulin heavy variable 7-4-1	0.1666029
AKR1C1	aldo-keto reductase family 1 member C1	0.16606572
MT1M	metallothionein 1M	0.16588064
IGKV2-24	immunoglobulin kappa variable 2-24	0.16414608
AL356585.2	immunoglobulin superfamily, member 3 (IGSF3) pseudogene	0.16320188
IGHV3-53	immunoglobulin heavy variable 3-53	0.16137895
IGLV2-14	immunoglobulin lambda variable 2-14	0.16123892
PNCK	pregnancy up-regulated nonubiquitous CaM kinase	-0.2791257
FOLH1B	folate hydrolase 1B	-0.25824046
AP000350.6	novel transcript	-0.24557075

SULT1C2	sulfotransferase family 1C member 2	-0.2387052
LINC01595	long intergenic non-protein coding RNA 1595	-0.22669141
CR2	complement C3d receptor 2	-0.22230598
SLC11A2	solute carrier family 11 member 2	-0.21172856
LINC02520	long intergenic non-protein coding RNA 2520	-0.20646778
PRODH	proline dehydrogenase 1	-0.20181172
SLC44A5	solute carrier family 44 member 5	-0.19921358
BTNL2	butyrophilin like 2	-0.19694428
HLA-DRB5	major histocompatibility complex, class II, DR beta 5	-0.19600105
AKR1B15	aldo-keto reductase family 1 member B15	-0.19437337
FRMD1	FERM domain containing 1	-0.18639672
NR1D1	nuclear receptor subfamily 1 group D member 1	-0.18151614
REC8	REC8 meiotic recombination protein [	-0.1803087
AC092071.1	novel transcript	-0.17963253
RNF207	ring finger protein 207	-0.17823352
HLA-DQB1	major histocompatibility complex, class II, DQ beta 1	-0.17815894
NCCRP1	non-specific cytotoxic cell receptor protein 1 homolog (zebrafish)	-0.17740272
MIF-AS1	MIF antisense RNA 1	-0.17701289
AC120498.6	novel transcript, antisense to CACNA1H	-0.17695062

PILRB	paired immunoglobin like type 2 receptor beta	-0.17612934
AC008676.3	Novel protein	-0.17195249
C17orf97	chromosome 17 open reading frame 97	-0.17002968
SPANXN3	SPANX family member N3	-0.16830634
XIST	X inactive specific transcript	-0.16704033
CACNA1H	calcium voltage-gated channel subunit alpha1 H	-0.16587123
AL645608.2	novel transcript	-0.16582304
LINC01235	long intergenic non-protein coding RNA 1235	-0.16561514
ADARB2	adenosine deaminase RNA specific B2 (inactive)	-0.16389473
SULT1C2P1	sulfotransferase family 1C member 2 pseudogene 1	-0.16342062
AP006621.5	TEC	-0.1566172
LINC00939	long intergenic non-protein coding RNA 939	-0.1559649
HERC2P3	hect domain and RLD 2 pseudogene 3	-0.15550421
TNFSF15	TNF superfamily member 15	-0.1548022
AC024580.1	TEC	-0.15327245
SMIM5	small integral membrane protein 5	-0.15283845
ZNF415	zinc finger protein 415	-0.15211613
NOS2	nitric oxide synthase 2	-0.15171868
AC021683.6	novel transcript	-0.15141359

SPNS1	sphingolipid transporter 1 (putative)	-0.15017784
AC126755.1	polycystic kidney disease 1 (autosomal dominant) (PKD1) pseudogene	-0.149649
AL033380.1	novel transcript	-0.14930837
PI4KAP1	phosphatidylinositol 4-kinase alpha pseudogene 1	-0.14839011
MROH7	maestro heat like repeat family member 7	-0.14632112
MUC20	mucin 20, cell surface associated	-0.14570752
ZNF300P1	zinc finger protein 300 pseudogene 1	-0.14477205
EHMT2-AS1	EHMT2 and SLC44A4 antisense RNA 1	-0.14440642
PSORS1C3	psoriasis susceptibility 1 candidate 3	-0.14281462

Degree of overrepresentation is indicated with ES (enrichment score), Positive ES indicates gene set enrichment at the top of the ranked list; a negative ES indicates gene set enrichment at the bottom of the ranked list.

Table S7. Correlation analysis between the plasma cell activation and the thickness of the intestinal glycocalyx.

	**Glycocalyx (nm)**	
n (pooled) = 16	$r_{Spearman}$=#, $r_{Pearson}$=†	***p***
**RER area in PC (μm^2)**	-0.72[#]	0.002
**Cytoplasm area in PC (μm^2)**	-0.55[#]	0.026
**RER area in PC cytoplasm (%)**	-0.77[†]	<0.001

**Number of mitochondria (average/PC)**	-0.56†	0.024

RER: rough endoplasmic reticulum; PC: plasma cell.

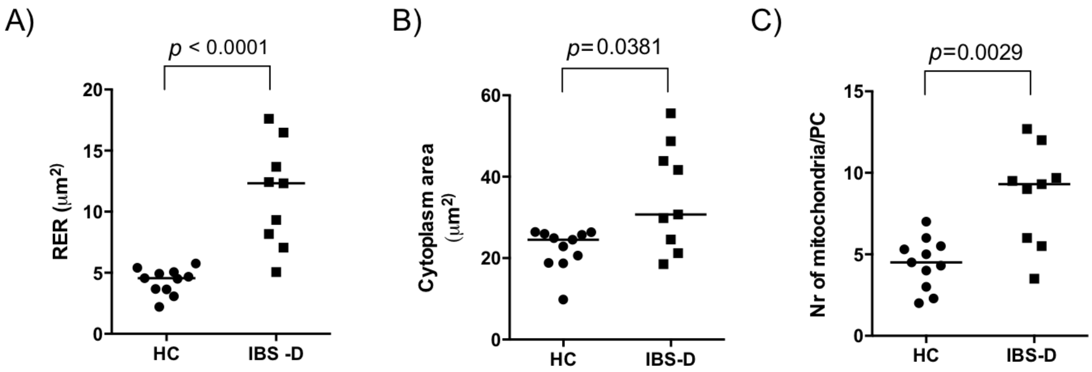

Figure S1. Quantification of plasma cell activation by morphometric analysis (A=RER, B=cytoplasm, C=number of mitochondria/PC) in IBS-D patients and HC. RER: rough endoplasmic reticulum; PC: plasma cell.

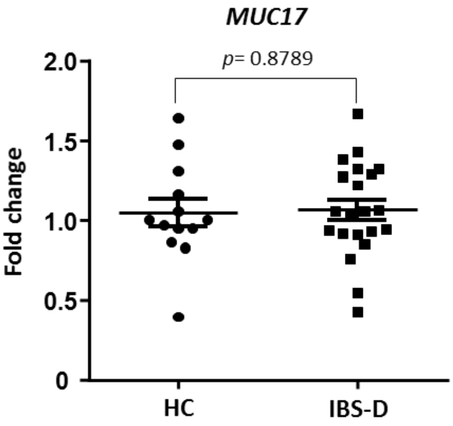

Figure S2. Quantitative gene expression of MUC 17 in the jejunal mucosa in HC and IBS-D groups. Individual values represent the fold change with respect to the average in the HC group. The 18S was used as a house-keeping gene. The *p* value is indicated (not significant).
